# Effects of aerobic exercise on vascular endothelial function and markers of oxidative stress and inflammation in individuals with impaired glucose tolerance: study protocol for a randomized controlled trial

**DOI:** 10.3389/fendo.2026.1828896

**Published:** 2026-04-30

**Authors:** Hancheng Wu, Zhouyu Liu, Dan Wang, Yiping Liu, Yuejia Li, Xuelu Li, Xiao Liu, Zhiwei Yan

**Affiliations:** 1 Provincial University Key Laboratory of Sport and Health Science, School of Physical Education and Sport Sciences, Fujian Normal University, Fuzhou, Fujian, China; 2 The First Affiliated Hospital of Dalian Medical University, Dalian, Liaoning, China; 3 Department of Breast Surgery and Oncology, The Second Hospital of Dalian Medical University, Dalian, Liaoning, China; 4 Cardiovascular and Metabolic Disorders Program, Duke-National University of Singapore Medical School, Singapore, Singapore; 5 Department of Cardiology, Sun Yat-sen Memorial Hospital of Sun Yat-sen University, Guangzhou, Guangdong, China

**Keywords:** aerobic exercise, endothelial function, impaired glucose tolerance, inflammation, oxidative stress

## Abstract

**Background and objective:**

Endothelial dysfunction is a key driver of cardiovascular complications in individuals with impaired glucose tolerance (IGT), with oxidative stress and inflammation playing pivotal roles in its pathogenesis. Although our previous studies have demonstrated that aerobic exercise (AE) significantly ameliorates IGT-induced endothelial dysfunction, the specific roles of oxidative stress and inflammation in this process remain to be elucidated. Therefore, we designed a randomized controlled trial to investigate the effects of AE on vascular endothelial function in patients with IGT and to further explore the underlying regulatory roles of oxidative stress and inflammation.

**Methods:**

Seventy individuals diagnosed with IGT will be enrolled in this two-arm, parallel-group randomized controlled trial, which utilizes an assessor-blinded design. Participants will be assigned in a 1:1 ratio to either a non-exercise control group or a 12-week supervised AE program, consisting of three weekly sessions at 65%–75% of their maximal heart rate. The primary endpoint will be the change in flow-mediated dilation (FMD) from baseline to week 12. Secondary endpoints will include cardiovascular function, glucose and lipid metabolism, circulating inflammatory and oxidative stress biomarkers, and *ex vivo* functional assays using participant-derived serum, including serum transfer experiments using human umbilical vein endothelial cells (HUVECs) and molecular profiling of endothelial progenitor cells (EPCs) cultured in the presence of the participants’ serum. Recruitment commenced in August 2025 and is currently ongoing.

**Discussion:**

This study aims to investigate the impact of a 12-week moderate-intensity AE intervention on vascular endothelial function, oxidative stress, and inflammation levels in patients with IGT. We hypothesize that, compared with the non-exercise control group, 12 weeks of AE will significantly improve endothelial function and exert both systemic and endothelial-derived antioxidant and anti-inflammatory effects.

**Clinical trial registration:**

https://www.chictr.org.cn/, identifier ChiCTR2500106827.

## 1 Introduction

Driven by changes in lifestyle and population aging, the prevalence of chronic metabolic diseases continues to increase, with impaired glucose tolerance (IGT) becoming increasingly common (1). IGT represents a high-risk transitional phase between normal glucose metabolism and type 2 diabetes mellitus (T2DM); it not only signals an elevated risk of developing diabetes but also serves as a potent predictor of cardiovascular disease (CVD) (2, 3). Epidemiological and clinical evidence suggest that vascular damage begins as early as the IGT stage (4, 5). Specifically, vascular endothelial dysfunction is recognized as a critical early event in the pathogenesis and progression of vascular disease. By reducing nitric oxide (NO) bioavailability, impairing vasodilatory responses, and promoting arterial stiffness and vascular remodeling, this dysfunction provides the fundamental pathological basis for adverse cardiovascular outcomes in individuals with IGT (6). Consequently, the targeted improvement of endothelial function constitutes a vital intervention strategy for mitigating cardiovascular risk in this population.

Oxidative stress and inflammation are considered primary drivers of metabolic dysregulation and endothelial dysfunction in patients with IGT (2, 7). Under physiological conditions, the body maintains redox homeostasis by regulating the generation and scavenging of reactive oxygen species (ROS) through endogenous antioxidant systems. However, in the context of IGT, glycemic fluctuations and insulin resistance can induce excessive ROS production, overwhelming endogenous antioxidant defenses, and ultimately leading to oxidative stress, metabolic disturbances, and endothelial dysfunction (8). Previous studies have demonstrated that the administration of antioxidants can improve endothelial function in patients with IGT, further highlighting the significance of antioxidant strategies for this population (9). In addition to oxidative stress, accumulating evidence from cellular and animal models of glucose dysregulation indicates that elevated ROS levels are also closely associated with the activation of inflammatory responses, which further aggravate endothelial injury (10–12). Therefore, strategies aimed at specifically modulating oxidative stress and inflammatory processes may represent an important approach for improving endothelial function in individuals with metabolic disorders, including IGT.

It is well established that aerobic exercise (AE) is a cornerstone of IGT management, promoting metabolic and cardiovascular health through various endogenous protective mechanisms. Our previous clinical and preclinical studies have demonstrated that AE ameliorates IGT-related vascular endothelial dysfunction by regulating the eNOS–NO signaling axis (13, 14). However, because of the practical challenges in directly obtaining vascular endothelial tissue from patients in a clinical setting, empirical evidence regarding whether AE-mediated endothelial improvement is linked to the regulation of oxidative stress and inflammation remains scarce. Two experimental paradigms—the use of exercise-conditioned serum and the *in vitro* analysis of endothelial progenitor cells (EPCs)—have provided feasible approaches to addressing this issue. These methods have been successfully employed in cardiovascular disease populations to explore the potential molecular mechanisms by which exercise interventions improve endothelial function (15, 16). Building on these experimental techniques, the present study aims to integrate exercise-conditioned serum experiments with *in vitro* EPC analysis. We will treat human umbilical vein endothelial cells (HUVECs) with serum collected from IGT patients before and after the intervention, while EPCs will be isolated and cultured from peripheral whole blood obtained from the same participants at the corresponding time points. This strategy will allow for a systematic evaluation of oxidative stress and inflammatory responses in endothelial cells, thereby exploring the potential mechanisms by which AE improves endothelial function in individuals with IGT.

We hypothesize that a 12-week moderate-intensity AE intervention will significantly improve vascular endothelial function in patients with IGT. Furthermore, AE may exert both circulating and endothelial-derived antioxidant and anti-inflammatory effects.

## 2 Materials and methods

### 2.1 Research design

This study will adopt a single-site, assessor-blinded, two-arm parallel controlled trial (RCT) design. Following screening, eligible patients with IGT will be randomly allocated to either the aerobic exercise (AE) group or the control (CON) group in a 1:1 ratio. Participants in the AE group will complete 12 weeks of supervised AE sessions (three times per week), whereas those in the CON group will be required to maintain their habitual lifestyle. Outcome assessments will be performed at baseline and at week 12 (post-intervention); the comprehensive schedule of enrollment, interventions, and assessments is detailed in **Table 1**. The study protocol has received ethical approval from the Ethics Committee of Fujian Normal University (No. 2025-5-30) and was registered with the Chinese Clinical Trial Registry (Identifier: ChiCTR2500106827). All participants will be required to sign a written consent form prior to enrollment. This trial will be reported in accordance with the Consolidated Standards of Reporting Trials (CONSORT) guidelines.

**Table 1:** Participant timeline: schedule of enrollment, interventions, and assessments.

Trial period:	Enrollment		Post-randomization	Close-out
Timepoint	Screening (day-14 to 0)	Baseline (day0)	Intervention period (weeks 1-12)	Week 12 (± 3 days)
Enrollment:				
Eligibility screen	X			
Informed consent	X			
Medical history	X			
Randomization		X		
Interventions:				
Aerobic Exercise Group			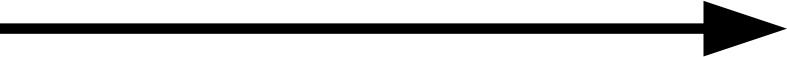	
Control Group			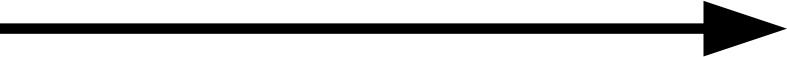	
Assessments:				
Primary outcomes				
Flow-mediated Dilation		X		X
Secondary outcomes				
Cardiovascular Function		X		X
Glucose and Lipid Metabolism		X		X
Circulating Biomarkers of Inflammation and Oxidative Stress		X		X
Human Umbilical Vein Endothelial Cells Serum Transfer Experiment		X		X
Endothelial Progenitor Cells Related Molecular Indicators		X		X
Safety monitoring				
Adverse Events		X	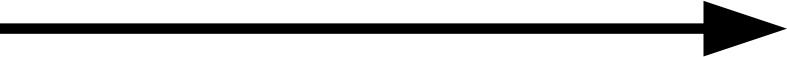	X

### 2.2 Research setting

**Figure 1:**
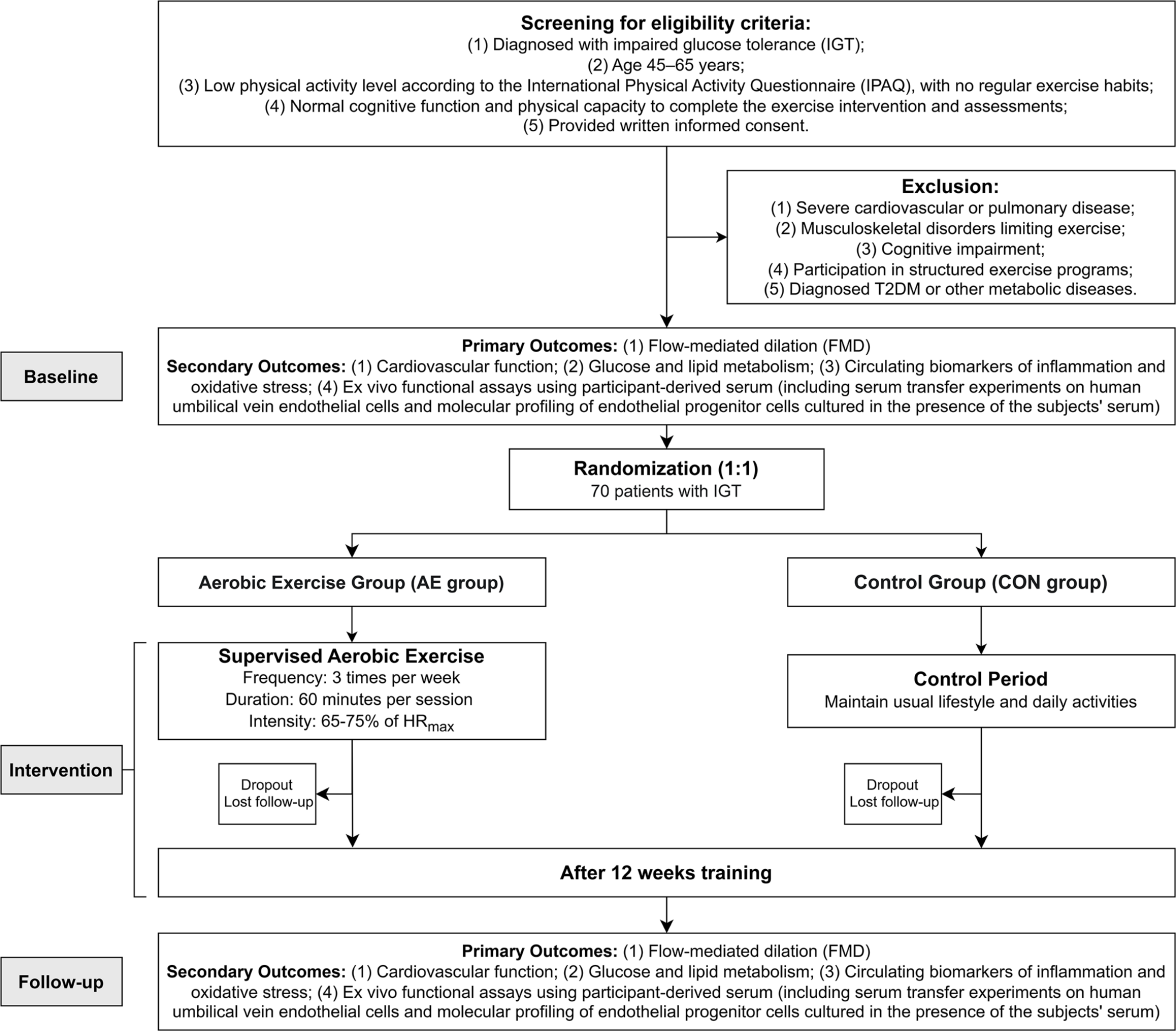
Flowchart of study procedures.

This study will be conducted in Fuzhou, Fujian Province. The research team will collaborate with community health service centers and clinics to recruit 70 patients with IGT through strategies including posting recruitment flyers, distributing promotional materials, and conducting health education lectures. All potential participants will undergo initial screening by community physicians, followed by further evaluation and confirmation by endocrinologists from tertiary hospitals and clinical exercise physiologists according to standardized inclusion and exclusion criteria. The exercise intervention will be implemented at exercise rehabilitation centers located within community health service centers, whereas clinical and functional assessments will be conducted at laboratories in partner hospitals and universities. To ensure rigorous adherence to the study protocol, specific roles and responsibilities have been assigned within the research team. Blinded researchers not involved in the recruitment process will be responsible for collecting baseline and post-intervention data. Clinical exercise physiologists not involved in recruitment or clinical assessment will conduct and supervise the exercise sessions. Recruitment was initiated in August 2025 and is currently ongoing (see **Figure 1**).

### 2.3 Inclusion criteria

Participants must meet all of the following criteria: (1) diagnosis of IGT, specifically defined as: a 2-h postload plasma glucose (2-h PG) level ≥ 7.8 mmol/L and < 11.1 mmol/L as confirmed by a 75-g oral glucose tolerance test (OGTT), with a fasting plasma glucose (FPG) level < 7.0 mmol/L (17); (2) age between 45 and 65 years; (3) identified as a “low physical activity level” via the International Physical Activity Questionnaire (IPAQ), with no regular exercise history; (4) normal cognitive function and the physical capacity necessary to complete the exercise intervention and clinical assessments; and (5) voluntary participation in the study with signed written informed consent.

### 2.4 Exclusion criteria

Candidates will be excluded from the trial if any of the following conditions are present: (1) diagnosis of severe cardiac disease, pulmonary dysfunction, or other medical conditions constituting contraindications to exercise; (2) confirmed musculoskeletal disorders resulting in mobility impairment; (3) cognitive or consciousness impairments; (4) current participation in other organized physical activity programs or formal sports training; and (5) diagnosis of T2DM or other metabolic diseases.

### 2.5 Sample size calculation

The sample size estimation for this study is based on the primary outcome, flow-mediated dilation (FMD). According to a previous systematic review and meta-analysis regarding the effects of exercise interventions on FMD in patients with T2DM (which included 10 randomized controlled trials and reported a mean FMD improvement of 1.77%, 95% CI: 0.94%–2.59%), the expected intergroup difference (δ) was set at 1.77% with a pooled standard deviation (σ) of 2.2% (18). In the absence of effect size estimates specifically derived from individuals with IGT, data from patients with T2DM were used as a reference. Previous studies have indicated that exercise-induced improvements in endothelial function may be attenuated in patients with T2DM compared to non-diabetic individuals (18). Therefore, using effect sizes derived from T2DM populations is considered a conservative assumption, which is unlikely to overestimate the intervention effect. Sample size calculation was performed using PASS software (NCSS, LLC, Kaysville, Utah, USA). Using a two-independent-sample t-test with a two-sided significance level (α) of 0.05 and a target statistical power ( 1−β) of 85%, the results indicated that 28 participants per group were required, totaling 56 participants. At this sample size, the actual statistical power to detect intergroup differences is 85%. Accounting for a potential dropout rate of 20%, we determined a total sample size of 70 participants. These participants will be randomly allocated to the two groups in a 1:1 ratio, with 35 participants recruited per group.

### 2.6 Random assignment and blinding

Eligible participants will be randomly assigned to either the AE group or the CON group in a 1:1 ratio. An independent researcher not involved in recruitment or data collection will generate the randomization sequence using Microsoft Excel. To ensure strict allocation concealment, group assignments will be placed in sequentially numbered, opaque, sealed envelopes (SNOSE), which will be maintained by a designated research assistant. To determine group assignment, the envelopes will be opened according to the order of enrollment only after participants have completed all baseline assessments.

This study utilizes an assessor-blinded randomized controlled design. Upon enrollment, participants will be assigned a unique identification code to ensure de-identified data management. All outcome measures, including anthropometric, imaging, and laboratory parameters, will be evaluated by blinded independent researchers. These assessors will remain unaware of both group assignments and specific measurement time points; furthermore, they will be strictly prohibited from communicating with participants regarding intervention experiences or grouping details. Similarly, independent statisticians will analyze de-identified datasets. Throughout the study, only the Data and Safety Monitoring Board (DSMB) and the interventionists (clinical exercise physiologists) will have access to group allocation data. All assessors and statisticians will remain strictly blinded until the final analysis is completed.

### 2.7 Dietary and medication control

To minimize potential confounding effects of diet and medication on metabolic, inflammatory, and oxidative stress biomarkers, stringent standardized controls will be implemented across the intervention period. Subjects will be instructed to preserve their habitual dietary patterns and will be prohibited from independently initiating any new dietary interventions, such as caloric restriction or specialized dietary regimens. Furthermore, participants will be required to limit alcohol consumption and maintain a consistent daily caffeine intake. At baseline, all medications and supplements that may influence glucose and lipid metabolism, inflammation, or oxidative stress—including hypoglycemic agents, lipid-lowering drugs, anti-inflammatory agents, and antioxidants—will be comprehensively documented. Subjects will be required to adhere to their existing medication regimens for the duration of the study. The use of any new medications or nutritional supplements will be prohibited unless prescribed by a physician. Any pharmacological changes during the study, including adjustments to medication type or dosage, will be accurately recorded.

### 2.8 Intervention

The intervention protocol for this study was developed with reference to the American College of Sports Medicine (ACSM) guidelines for prescribing exercise to T2DM populations (19). All supervised sessions will be scheduled 1–2 hours postprandially to manage exercise-related glucose fluctuations. Given that the acute enhancement of insulin sensitivity following a single exercise bout typically persists for only 24–72 hours, the interval between consecutive sessions will be maintained at no more than 48 hours to ensure cumulative metabolic benefits and prevent the attenuation of intervention effects (19, 20). The AE intervention will follow the FITT principles (frequency, intensity, time, type) and will be managed by exercise physiologists. The intervention period will span 12 weeks, with three supervised sessions per week. Each session will consist of a 5-minute preparatory warm-up (50%–60% of HR_max_), 50 minutes of aerobic exercise (65%–75% of HR_max_), and a 5-minute cool-down (50%–60% of HR_max_). The HR_max_ will be calculated using the Tanaka formula: HR_max_ =208−0.7×age. Participants will utilize heart rate monitors (Polar Electro, Kempele, Finland) for instantaneous intensity observation to ensure exercise intensity remains within the target range. For participants exhibiting chronotropic incompetence (blunted heart rate response) or those receiving heart rate-modulating medications (e.g., non-dihydropyridine calcium channel blockers or β-blockers), exercise intensity will be prescribed using a combination of the Borg Rating of Perceived Exertion (target range 12–14) and heart rate. The control (CON) group will receive no structured exercise intervention and will receive clear directions to maintain their habitual lifestyle. During the intervention period, researchers will assess the physical activity levels of the CON group every two weeks using the International Physical Activity Questionnaire (IPAQ). Participants in the intervention group will also be instructed not to engage in any additional structured exercise beyond the prescribed training program during the study period to ensure consistency of the intervention dose. Considering the minimum effective dose required for exercise-induced benefits, participants who complete less than 75% of the prescribed training sessions will be considered non-adherent in the per-protocol analysis.

### 2.9 Safety considerations and adverse event monitoring

Before each training session, a mandatory health evaluation will be performed to screen for symptoms including excessive fatigue, palpitations, and dizziness. We will perform finger-prick glucose tests exclusively on participants showing signs of acute dysregulation (e.g., diaphoresis, tremors, or lightheadedness) or general malaise. The exercise intervention will be postponed if blood glucose levels meet any of the following criteria: > 16.7 mmol/L; < 3.9 mmol/L; or > 13.9 mmol/L accompanied by symptoms of metabolic decompensation or ketonuria.

In accordance with ACSM standards, sessions will be terminated immediately upon detecting angina-like symptoms; a decrease in systolic blood pressure (SBP) > 10 mmHg despite an increase in intensity; or an exaggerated hypertensive response (diastolic blood pressure [DBP] >115 mmHg or SBP > 250 mmHg); signs of poor perfusion, dizziness, or severe shortness of breath; and pronounced ST-segment changes or clinically significant arrhythmias. All adverse events—including cardiovascular symptoms, musculoskeletal injuries, and any unexpected reactions—will be formally recorded by the supervising exercise physiologist in a standardized Case Report Form (CRF). These records will be independently adjudicated by a DSMB, which will evaluate the severity and causality of the events to the intervention to decide on the necessity of trial modifications.

### 2.10 Measurements

Data collection will be performed by blinded researchers independent of the recruitment process. Prior to the commencement of testing, all assessment personnel will undergo uniform standardized training to ensure the consistency and reliability of measurements. To guarantee strict adherence to the study protocol, participants will be provided with detailed information regarding the operating procedures, research objectives, and specific requirements before any measurements are taken. Assessments will be scheduled at baseline and at week 12 (post-intervention). The primary outcome will be FMD. Secondary outcomes will include cardiovascular function, glucose and lipid metabolism, circulating biomarkers of inflammation and oxidative stress, and *ex vivo* functional assays using participant-derived serum, including serum transfer experiments using HUVECs and molecular profiling of EPCs cultured in the presence of the participants’ serum. With the exception of the OGTT and the 2-h PPG test, all assessments will be conducted following a 12-h overnight fast. Subjects will be requested to avoid strenuous physical activity and abstain from caffeine and alcohol for 24 hours prior to evaluation. They will also be required to wear loose-fitting clothing. On the morning of the assessment, routine medications will be withheld to eliminate potential pharmacological interference. In order to eliminate the acute biological effects of a single exercise bout, we will schedule post-intervention tests after a two-day rest period (48 hours) following the last session. Furthermore, to avoid reactive hyperglycemia induced by prolonged fasting, venous blood sampling will be uniformly performed the morning following an overnight fast.

### 2.11 Blood collection and preparation

Following a 12-h overnight fast, approximately 8 mL of peripheral venous blood will be collected from participants via median cubital venipuncture. Blood samples will be distributed into EDTA-anticoagulated tubes for the measurement of glycated hemoglobin (HbA1c) and the isolation of PBMCs, and into serum collection tubes for serum preparation. Serum will be used for routine biochemical analyses (glucose and lipids), insulin measurement, assessment of circulating inflammatory and oxidative stress biomarkers, as well as for serum transfer experiments and the establishment of a human serum culture system for EPCs. After clotting at room temperature for 30 min, samples in serum collection tubes will be centrifuged at 3,000 × g for 15 min at 4 °C. The resulting serum will be collected under sterile conditions, filtered through a 0.22 μm syringe filter for cell culture applications, aliquoted to avoid repeated freeze–thaw cycles, and stored at −80 °C until analysis.

### 2.12 Primary outcome

#### 2.12.1 Vascular endothelial function

Vascular endothelial function will be assessed via flow-mediated dilation (FMD). Measurements will be conducted by professionally trained researchers utilizing an ultrasound vascular imaging unit (UNEXEF18G; UNEX, Japan). Measurement procedures will follow our previously published paper (21). The formula for FMD is as follows:


$$\text{FMD}\%=\frac{\text{peak post}-\text{hyperemic diameter }-\text{ baseline arterial diameter}}{\text{baseline arterial diameter}}\times 100\%.$$

### 2.13 Secondary outcomes

#### 2.13.1 Glucose metabolism

Glucose metabolism parameters will include fasting blood glucose (FBG), glucose levels during a standard 75-g oral glucose tolerance test (OGTT), 2-h postprandial glucose (2-h PPG), and glycated hemoglobin (HbA1c). An automated biochemical analysis system equipped with standardized enzymatic assays will be utilized to determine plasma glucose concentrations (FBG, 2-h PPG, and 2-h OGTT). Meanwhile, high-performance liquid chromatography (HPLC) will be employed for the quantification of HbA1c. Measurement procedures will follow our previously published paper (21).

#### 2.13.2 Insulin level

Fasting insulin levels will be determined using enzyme-linked immunosorbent assay (ELISA) kits. Subsequently, the Homeostatic Model Assessment of Insulin Resistance (HOMA-IR) index will be calculated in conjunction with FBG values to evaluate insulin sensitivity. The formula for HOMA-IR is as follows:


HOMA-IR=Fasting blood glucose (mmol/L)×Fasting insulin (µU/mL)/22.5

#### 2.13.3 Lipid metabolism

Lipid metabolism parameters will include total cholesterol (TC), triglycerides (TG), high-density lipoprotein cholesterol (HDL-C), and low-density lipoprotein cholesterol (LDL-C). Plasma concentrations of these lipids will be determined using an automated biochemical analyzer with the corresponding commercial kits.

#### 2.13.4 Cardiovascular risk factors

Body weight, standing height, hip circumference (HC), and waist circumference (WC) will be measured using a digital scale, a stadiometer, and a non-elastic tape measure, respectively. Body mass index (BMI) will be computed as body weight (kg)/height squared (m^2^), whereas the waist-to-hip ratio (WHR) will be determined as WC (cm)/HC (cm). Body fat distribution will be evaluated through a combination of skinfold thickness testing and ultrasound scanning. Skinfold thickness will be measured at the triceps, abdomen, and subscapular sites using a skinfold caliper, with readings recorded to the nearest 1 mm. Ultrasound scanning will be performed using a calibrated analyzer (BodyMetrix BX-2000; IntelaMetrix, USA) at the following seven sites: midaxillary, subscapular, triceps, abdomen, chest, suprailiac, and thigh. Measurement procedures will follow our previously published paper (21).

#### 2.13.5 Arterial stiffness

Arterial stiffness will be assessed via brachial-ankle pulse wave velocity (baPWV). Measurements will be performed by professionally trained researchers using an automated arteriosclerosis screening device (BP-203RPE III; Omron, Japan). Measurement procedures will follow our previously published paper (21).

#### 2.13.6 Blood pressure

Blood pressure (BP) will be measured by trained cardiologists using an automated blood pressure monitor (Omron HEM 3; Omron, Japan). Measurement procedures will follow our previously published paper (21).

#### 2.13.7 Circulating oxidative stress biomarkers

To characterize systemic oxidative stress status at the circulating level, biomarkers will be assessed in serum samples. Serum levels of ROS will be assessed using a fluorescence-based assay kit. Serum malondialdehyde (MDA) levels will be determined using a thiobarbituric acid (TBA)-based colorimetric assay (TBARS method), and superoxide dismutase (SOD) activity will be measured using a WST-1–based colorimetric assay, both performed using commercially available microplate-based kits. Additionally, serum concentrations of nuclear factor erythroid 2–related factor 2 (Nrf2) will be quantified using ELISA kits following standard protocols.

#### 2.13.8 Circulating inflammatory biomarkers

To reflect systemic inflammatory status at the circulating level, inflammatory biomarkers will be measured in serum samples. Serum levels of NLR family pyrin domain containing 3 (NLRP3), interleukin-1β (IL-1β), tumor necrosis factor-alpha (TNF-α), interleukin-6 (IL-6), high-sensitivity C-reactive protein (hs-CRP), and interleukin-10 (IL-10) will be quantified using ELISA kits following standard protocols.

#### 2.13.9 HUVEC serum transfer experiment and cellular analyses

To investigate the regulatory effects of exercise-induced circulating factors on endothelial redox homeostasis and inflammation, serum transfer experiments will be conducted using individual serum samples obtained from all participants in both the AE and CON groups. To preserve individual biological variability, serum from each participant will be applied individually to cell cultures without pooling. Human umbilical vein endothelial cells (HUVECs; Servicebio, Cat# STCC12103P-1) will be cultured to 70%–80% confluency, followed by synchronization in low-serum medium (0.5%–1% FBS) for 4–6 h. The cells will then be incubated for 24–48 h in medium supplemented with 2% individual participant serum. After the incubation period, the cells will be harvested for subsequent functional and molecular assessments.

To evaluate cellular redox homeostasis and inflammatory responses, intracellular ROS levels will be determined using a fluorescent probe assay. The protein expression of key oxidative stress–related molecules—including Nrf2, Kelch-like ECH-associated protein 1 (Keap1), and heme oxygenase-1 (HO-1)—and inflammatory mediators—including NLRP3, IL-10, interleukin-18 (IL-18), and IL-1β—will be assessed via Western blot analysis. Concurrently, the mRNA expression levels of HO-1 will be quantified using RT-qPCR, and relative gene expression will be calculated using the 2^−ΔΔCt^ method. Detailed protocols for the measurement of intracellular ROS levels, protein extraction, Western blot analysis, RNA extraction, and RT-qPCR are provided in the **Supplementary Material**.

#### 2.13.10 EPC culture under human serum conditions and cellular analyses

To further investigate whether exercise confers endothelial protection by modulating EPC function, EPC-related analyses will be conducted using peripheral blood samples collected from all participants in both the AE and CON groups. EPCs will be cultured and maintained within a human serum–based system using serum obtained from the same participants at the corresponding time points, aiming to more closely mimic the *in vivo* circulating microenvironment while preserving inter-individual biological variability.

Anticoagulated peripheral blood samples will be diluted with phosphate-buffered saline (PBS), and peripheral blood mononuclear cells (PBMCs) will be isolated via density gradient centrifugation. The isolated cells will then be seeded onto fibronectin-coated culture plates and maintained at 37 °C in a humidified incubator with 5% CO_2_. The culture medium will consist of endothelial basal medium supplemented with 5% autologous human serum, without the addition of fetal bovine serum (FBS). Endothelial colony-forming units (CFU-EC/CFU-Hill colonies) will be identified and quantified between days 7 and 10 of culture as a functional index. These colonies will be identified based on their characteristic morphology, consisting of a central cluster of rounded cells surrounded by radiating spindle-shaped cells. For subsequent molecular analyses, the adherent EPCs derived from these colonies will be expanded. Upon reaching 80%–90% confluency, these cells will be subcultured, with early passages (P1 to P3) used to evaluate redox homeostasis and inflammatory markers. The molecular biomarkers to be evaluated in EPCs will be consistent with those assessed in the HUVEC experiments.

### 2.14 Statistical analysis

All statistical analyses will be conducted using R (version 4.3.1; R Foundation for Statistical Computing, Vienna, Austria). For descriptive statistics of baseline characteristics, normally distributed continuous variables will be expressed as mean±standard deviation (SD), whereas non-normally distributed data will be presented as median (interquartile range [IQR]). Categorical variables will be expressed as frequencies and percentages [n (%)]. Intergroup comparisons of baseline characteristics will be performed using independent t-test or Mann–Whitney U test for continuous data, and Chi-square (χ^2^) or Fisher’s exact test for categorical data, as appropriate. Efficacy analyses will follow the intention-to-treat (ITT) principle, including all randomized participants. To evaluate intervention effects between baseline and week 12, a linear mixed-effects model (LMM) will be employed. In this model, time (baseline and week 12), group (AE vs. CON), and the group×time interaction term will be included as fixed effects, while participants will be treated as a random effect (random intercept) to account for the intra-individual correlation arising from repeated measurements. Primary inferences will be based on the group×time interaction term to determine whether the change over time differs significantly between the two groups. If the interaction term reaches statistical significance, further *post hoc* comparisons will be performed to examine intergroup differences at week 12 and intragroup changes from baseline to week 12. Model estimates will report estimated marginal means with their corresponding 95% confidence intervals (CIs). Since LMMs provide unbiased estimates under the missing at random (MAR) assumption by utilizing all available data, no additional data deletion will be performed. As a sensitivity analysis, a per-protocol (PP) analysis will be conducted, including only participants who completed ≥ 75% of the prescribed training sessions and had no major protocol deviations. Furthermore, sensitivity analyses for all outcome measures will be further adjusted for potential confounders, such as age and sex, to verify the robustness of the findings. The primary outcome will be used for confirmatory inference. Secondary outcomes will be considered exploratory, and no formal adjustment for multiple comparisons will be applied; therefore, these results will be interpreted with caution, with emphasis on effect sizes and consistency of findings. All statistical tests will be two-sided, and *P* < 0.05 will be considered statistically significant.

## 3 Discussion

Individuals with IGT, representing an early stage in the progression of metabolic diseases, are a critical target for intervention. AE is recognized as a cornerstone lifestyle intervention capable of enhancing metabolic function. While extensive research has established that AE significantly mitigates the risk of IGT progressing to T2DM, evidence regarding its efficacy in improving specific glycemic parameters in patients with IGT remains inconsistent (22, 23). To address this, the present study will systematically evaluate the potential effects of AE on the glycemic status of patients with IGT by assessing FBG, 2-h PPG, HbA1c, OGTT performance, and insulin resistance levels. Furthermore, central obesity—characterized by abdominal adipose accumulation—is intricately linked to the metabolic disturbances and cardiovascular risks associated with IGT (24, 25). Therefore, this study will also employ waist-to-hip ratio (WHR) measurements, bioelectrical impedance analysis, and ultrasound imaging to assess abdominal fat distribution, allowing for a deeper exploration of the impact of AE on central obesity and regional fat deposition in the IGT population.

Beyond metabolic impacts, vascular structural and functional impairments represent critical pathological features of IGT. Previous research indicates that individuals with IGT already exhibit a discernible predisposition toward atherosclerosis, with endothelial dysfunction recognized as a pivotal early event in this process (26, 27). Nevertheless, clinical evidence regarding the influence of AE on the atherosclerotic process specifically in patients with IGT remains relatively limited. Consequently, the present study will employ FMD and baPWV to assess the impact of AE on vascular endothelial function and the severity of arterial stiffness in this population.

Oxidative stress and inflammation are recognized as fundamental mechanisms driving IGT-associated endothelial dysfunction and the development of atherosclerosis (7, 28). However, due to the inherent challenges in directly obtaining vascular endothelial tissue from patients in a clinical setting, empirical evidence regarding whether AE-mediated endothelial protection is achieved through the modulation of oxidative stress and inflammatory responses remains scarce. The exercise-conditioned serum technique—which involves applying human serum collected before and after an exercise intervention to cell cultures—enables the observation of how exercise-associated circulating factors regulate cellular phenotypes and signaling pathways. This approach has been widely implemented in exercise-related endothelial biology research (15). Furthermore, as precursors to endothelial cells, EPCs play a crucial role in reflecting vascular repair capacity and endothelial function; notably, individuals with IGT frequently exhibit impaired EPC mobilization and functional deficits (29). Building on this rationale, the present study will integrate exercise-conditioned serum experiments with *in vitro* EPC analysis. HUVECs will be treated with serum collected from IGT patients before and after the intervention, while EPC-related markers will be evaluated in parallel. The effects of AE on endothelial oxidative stress will be assessed by measuring intracellular ROS levels and antioxidant-related markers, including Nrf2, and HO-1. Inflammatory responses will be evaluated by quantifying pro-inflammatory mediators (NLRP3, IL-18, and IL-1β) and the anti-inflammatory cytokine IL-10. Moreover, given the systemic impact of oxidative stress and inflammatory responses on metabolic and vascular health, this study will also quantitatively assess circulating biomarkers of oxidative stress (SOD, Nrf2, ROS, and MDA) and inflammation (NLRP3, TNF-α, IL-6, IL-10, hs-CRP, and IL-1β). This approach will provide a comprehensive evaluation of how AE modulates the redox status and inflammatory profile in the IGT population.

Consequently, this study will focus on the dual pathological hallmarks of oxidative stress and inflammation. By evaluating endothelial function in conjunction with two distinct experimental paradigms—exercise-conditioned serum and EPC analysis—we will seek to conduct an in-depth investigation into the potential mechanisms underlying AE-mediated endothelial protection. This research is designed to bridge the existing gap between basic physiological mechanisms and organ-scale functional performance. By integrating clinical evidence with a multidimensional mechanistic exploration spanning the “molecule-cell-organ” hierarchy, this study will provide novel insights and a theoretical basis for exercise prescriptions tailored to patients with IGT.

## 4 Limitations

Certain drawbacks of the current research design need to be acknowledged. First, a primary objective of precision exercise prescription is to define the dose-response relationship between various exercise doses and health outcomes. However, due to constraints in study design and practical feasibility, the present trial will focus on a single exercise intensity and modality, precluding a comprehensive evaluation of how different exercise volumes might influence the study outcomes. Second, the applicability of the results to different ethnicities or regions may be restricted, given that this is a single-center study focused on Chinese community-dwelling adults. Third, owing to limitations in experimental resources and staffing, a long-term follow-up will not be incorporated into the design; therefore, the sustainability of exercise-induced benefits remains to be determined. Finally, the inclusion of multiple secondary and exploratory outcomes may increase the risk of Type I errors. Therefore, we will interpret the data from these exploratory variables as hypothesis-generating rather than definitive. Nonetheless, this study is expected to provide a robust theoretical foundation and clinical translational potential for promoting vascular health in the IGT population from both clinical and mechanistic perspectives.
