# Supplementary material for: Effects of aerobic exercise on vascular endothelial function and markers of oxidative stress and inflammation in individuals with impaired glucose tolerance: study protocol for a randomized controlled trial
Source: Front Endocrinol (Lausanne). 2026 Apr 30;17:1828896. doi: 10.3389/fendo.2026.1828896 (PMC13171376; doi:10.3389/fendo.2026.1828896)
Supplement: Supplementary file 1 [file DataSheet1.docx]

Supplementary Material

# Measurement of intracellular reactive oxygen species levels

Intracellular reactive oxygen species (ROS) levels will be determined using a 2',7'-dichlorodihydrofluorescein diacetate (DCFH-DA) fluorescent probe. Following the serum treatments, the culture medium will be discarded and replaced with DCFH-DA working solution (10 μmol/L in serum-free medium), followed by incubation at 37 ^°^C for 20 min in the dark. After incubation, the cells will be washed three times with PBS to remove any extracellular probe. Fluorescence intensity will then be detected using a confocal microscope. ROS levels will be expressed as the mean fluorescence intensity (MFI) per unit area, with all images captured and quantified using consistent exposure parameters. Each experiment will include technical replicates and be repeated at least three times independently.

# Protein extraction and Western blot analysis

Total cellular proteins will be extracted using a lysis buffer supplemented with protease and phosphatase inhibitor cocktails. Protein concentrations will be determined via the bicinchoninic acid (BCA) assay, after which samples will be normalized to equal concentrations and denatured at 95 ^°^C for 5 min. Equal amounts of protein will be separated by sodium dodecyl sulfate-polyacrylamide gel electrophoresis (SDS-PAGE) and subsequently transferred onto polyvinylidene fluoride (PVDF) membranes. The membranes will be blocked with 5% non-fat dry milk for 1–2 h and then incubated with specific primary antibodies overnight at 4 ^°^C. After being washed three times with Tris-buffered saline containing Tween-20 (TBST) for 10 min each, the membranes will be incubated with a horseradish peroxidase (HRP)-conjugated secondary antibody for 1 h at room temperature. Following another set of washes, protein bands will be visualized using enhanced chemiluminescence (ECL) reagents. Densitometric analysis of the band signals will be performed using ImageJ software (NIH, Bethesda, MD, USA). The relative expression levels of target proteins will be normalized to internal controls and expressed as fold changes relative to the control group.

# RNA extraction and RT-qPCR analysis

Total cellular RNA will be isolated using an RNA extraction kit. The concentration and purity of the extracted RNA (A_260_/A_280_ ratio) will be assessed using a spectrophotometer. Subsequently, the RNA will be reverse-transcribed into cDNA using a reverse transcription kit. Quantitative real-time PCR (qPCR) will be performed with the cDNA template using the SYBR Green fluorescent dye system on a real-time PCR system. Each 20 μL reaction mixture will comprise 2 × qPCR Mix, specific forward and reverse primers, and the cDNA template. The thermal cycling program will be set as follows: initial denaturation at 95 ^°^C for 30 s, followed by 40 cycles of denaturation at 95°C for 5 s and annealing/extension at 60 ^°^C for 30 s. Upon completion of the amplification, a melting curve analysis will be conducted to confirm the specificity of the PCR products. Relative gene expression levels will be calculated using the 2^−ΔΔCt^ method. All experiments will be performed in technical triplicates and repeated in at least three independent experiments.
